# The role of immune cells in Hantaan-induced hemorrhagic fever with renal syndrome

**DOI:** 10.3389/fimmu.2026.1921852

**Published:** 2026-07-28

**Authors:** Lin Su, Yinli He, Linpei Zhang, Yawen Wang, Xiaojiao Li, Minhong Fei

**Affiliations:** 1 Infectious Diseases Department, The First Affiliated Hospital of Xi’an Jiaotong University, Xi’an, Shaanxi, China; 2 BioBank, The First Affiliated Hospital of Xi’an Jiaotong University, Xi’an, Shaanxi, China; 3 Shaanxi Engineering Research Center for Biobank and Advanced Medical Research, The First Affiliated Hospital of Xi’an Jiaotong University, Xi’an, Shaanxi, China; 4 Department of Laboratory Medicine, First Affiliated Hospital, School of Medicine, Xi’an Jiaotong University, Xi’an, Shaanxi, China; 5 Jiangyan Hospital of Traditional Chinese Medicine, Taizhou, Jiangsu, China

**Keywords:** dynamic changes, HFRS, HTNV, immune cells, regulatory mechanisms

## Abstract

Hemorrhagic fever with renal syndrome (HFRS) is a severe and often fatal zoonotic disease primarily caused by the Hantaan virus (HTNV), a member of the Orthohantavirus genus. Although immune cells are known to play critical roles in HTNV-induced HFRS, the detailed dynamic changes, phenotypic alterations, and functional profiles of distinct immune subsets over the course of the disease remain poorly understood. In this review, we summarize current understanding of key immune populations during HTNV infection, including monocytes, macrophages, dendritic cells (DCs), natural killer (NK) cells, neutrophils, T cells and B cells. We highlight a central immunological duality in which these cells are necessary for viral clearance while also participating in immunopathological tissue damage. By integrating clinical and mechanistic insights, we present a detailed framework for understanding how the balance between protective immunity and immunopathology determines disease outcome. Recognizing these complex dynamics will be crucial for directing future studies and determining potential therapeutic targets in HFRS.

## Introduction

Hantaviruses belong to the family Hantaviridae within the order Bunyavirales. They possess a tripartite negative-sense single-stranded RNA genome, consisting of large (L), medium (M), and small (S) segments. These segments encode an RNA-dependent RNA polymerase, envelope glycoproteins Gn and Gc, and the nucleocapsid protein, respectively (1). The viral particles are spherical, approximately 80 to 120 nm in diameter, and assemble and bud at the Golgi complex. To date, the International Committee on Taxonomy of Viruses has officially recognized 24 hantavirus species. These viruses are transmitted to humans through rodent hosts and are distributed worldwide. However, many hantaviruses probably remain undiscovered and unreported in regions such as Africa, the Middle East, and the Indian subcontinent (1).

Hantavirus infection in humans typically leads to two clinical syndromes, hemorrhagic fever with renal syndrome (HFRS) and hantavirus cardiopulmonary syndrome (HCPS). The viruses causing HFRS mainly include Hantaan virus (HTNV) in Asia, Puumala virus (PUUV) and Dobrava virus (DOBV) in Europe, and Seoul virus (SEOV) with a global distribution. The viruses responsible for HCPS include Sin Nombre virus (SNV) in North America and Andes virus (ANDV) in Latin America (2). Globally, HFRS leads to about 150,000 to 200,000 infections annually, with a case fatality rate ranging from 5% to 15% (1, 3). China is the most highly endemic region for HFRS, contributing over 90% of the total cases worldwide. Among these pathogenic hantaviruses, HTNV is the primary causative agent of HFRS in China (4). HTNV-induced HFRS clinically presents with fever, hemorrhage, and renal dysfunction.

Notably, the pathogenesis of HTNV cannot be solely attributed to direct viral cytotoxicity. *In vitro* investigations have shown that HTNV infection fails to induce significant cytopathic effects or extensive apoptosis in confluent cell monolayers, which serve as models for endothelial and epithelial cells (5). This finding suggests that viral replication alone cannot explain the severe vascular leakage and organ injury observed in HTNV-induced HFRS patients. This notion is further supported by recent longitudinal monitoring of HTNV RNA kinetics, which demonstrates that the viral load during the febrile phase​ is a strong indicator of disease severity, yet the duration of viremia shows no correlation with clinical outcomes (6). These observations imply that there are mechanisms other than direct viral replication that contribute to disease severity.

In fact, it has been well-documented that HTNV can affect a wide range of immune cells, including monocytes and macrophages (7–10), dendritic cells (DCs) (11, 12), natural killer (NK) cells (13–17), neutrophils (18, 19), T cells (20–24), and B cells (25–27). Upon infection, these cells exhibit marked phenotypic and functional changes. For example, monocytes polarize towards the M1 macrophages (10), DCs upregulate co-stimulatory molecules (12), NK cells display an activated and proliferative phenotype with enhanced cytokine secretion (16, 28), neutrophils exhibit myeloperoxidase (MPO) release and neutrophil extracellular trap (NET) formation (18, 29), and T and B cells undergo proliferation and effector differentiation (14, 26, 30, 31). Moreover, studies have shown that HTNV infection induces the release of multiple pro-inflammatory cytokines and chemokines, such as CXCL10, IL-33, and CX3CL1, whose plasma levels positively correlate with disease severity (32–35). Taken together, these observations indicate that the host immune system is actively engaged and likely plays a central role in the pathogenesis of HTNV-induced HFRS.

Despite these advances, a comprehensive understanding of the dynamic changes, phenotypic alterations, and functional profiles of immune cell subsets across HTNV disease stages is still lacking. The regulatory mechanisms that control immune activation and contraction are not well understood. In this review, we aim to systematically assemble the existing evidence on these immune cell characteristics and their underlying regulatory networks. By integrating recent findings from clinical, molecular, and animal studies, we aim to provide a comprehensive framework that highlights both established patterns and unresolved mechanistic questions, thereby serving as a foundation for future investigations.

## Macrophage activation and subset dysregulation drive HFRS pathogenesis and host species differences

HTNV starts its infection by preferentially targeting CD14 positive monocytes and myeloid dendritic cells (cDCs) within the peripheral blood mononuclear phagocyte system (MPS) (7). Among human peripheral blood monocytes, three subsets are defined based on CD14 and CD16 expression: classical (CD14^++^CD16^-^), intermediate (CD14^++^CD16^+^), and nonclassical (CD14^+^CD16^++^) (36). Studies by Li and colleagues have confirmed that HTNV predominantly infects and replicates in classical and intermediate monocyte subsets, which are collectively termed inflammatory monocytes (9). This selective infection triggers a robust pro-inflammatory response in MPS cells, characterized by increased production of tumor necrosis factor-α (TNF-α), interleukin-1β (IL-1β), and interleukin-6 (IL-6) (8). Mechanistically, it has been demonstrated that HTNV triggers IL-1β secretion through activation of the leucine-rich repeat containing protein 3 (NLRP3) inflammasome and caspase-1-dependent cleavage of pro-IL-1β (37). Lee et al. also reported that the RNA generated during HTNV replication is recognized by the cytosolic sensor retinoic acid inducible gene-I (RIG-I), which in turn triggers the production of type I interferons (38). Recent evidence further shows that HTNV promotes M1-like monocyte/macrophage responses in humans via Notch–NF-κB signaling, leading to elevated levels of TNF-α and IL-6 (10). These findings demonstrate that HTNV infection of monocyte cells can elicit a robust inflammatory response.

Consistent with these observations, clinical evidence further supports sustained activation of the monocyte/macrophage compartment during HFRS. Studies have reported significantly elevated plasma levels of soluble CD14 (sCD14) and soluble CD163 (sCD163) in HFRS patients during the acute phase (39–41). Notably, plasma sCD163 levels are higher in severe/critical cases than that in mild/moderate cases (40). In addition, monocyte chemoattractant protein 1 (MCP-1) levels are also elevated and positively correlated with white blood cell count (WBC), blood urea nitrogen (BUN), and creatinine (Cr) levels, while negatively correlated with platelet (PLT) count (42). These findings suggest that sustained activation of the monocyte/macrophage plays an important role in the pathological process of HFRS.

To further examine whether specific monocyte subsets are responsible for the sustained activation observed in HFRS, recent studies have focused on changes at the monocyte subset level. During the acute phase of HFRS, the number of CD14^++^CD16^+^ intermediate monocytes in the peripheral blood is significantly increased compared with the convalescent phase and healthy controls, and their absolute counts are higher in severe/critical patients than in mild/moderate patients (9). These findings suggest that the number of intermediate monocytes can serve as a potential immunological marker for assessing the severity of HFRS. Beyond changes in cell number, the surface expression of CD163 and CD206 on these intermediate monocytes was also altered. Specifically, the frequency of CD14^++^CD16^+^CD163^+^ monocytes is positively correlated with WBC, BUN and Cr levels. In contrast, the frequencies of CD14^++^CD16^+^CD206^+^ monocytes are negatively correlated with PLT count and Cr level, but positively correlated with plasma IL-10 levels. Moreover, the frequency of CD14^++^CD16^+^IL-10^+^ monocytes is negatively correlated with PLT count and positively correlated with plasma IL-10 levels (9). These results indicate that during acute HFRS, the expansion of intermediate monocytes and their altered surface marker profiles are closely linked to disease severity and laboratory parameters.

In addition to changes in monocyte subset distribution, HTNV infection is associated with the emergence of CD226^−^ inflammatory monocytes with reduced antigen-presenting potential. Tang et al. identified a population of CD226^−^ inflammatory monocytes in the peripheral blood of acute HFRS patients, whose proportion was significantly higher than in convalescent patients or healthy individuals (43). Functionally, these cells exhibit low expression of human leukocyte antigen (HLA) -DR/DP/DQ and the co-stimulatory molecule CD80, indicating impaired antigen presenting capacity. Clinically, the proportion of these cells is positively correlated with plasma HTNV load and negatively correlated with PLT count. Supporting these findings, *in vitro*, CD226 overexpression enhanced HLA-DR/DP/DQ and CD80 expression in THP-1 cells, whereas HTNV infection reduced CD226, HLA-DR/DP/DQ, and CD80 expression in CD226-overexpressing THP-1-derived macrophages. Consistently, HTNV challenge in mice induced splenic CD226^−^ macrophage expansion with reduced I-A/I-E and CD80 expression, further supporting the notion that the CD226^−^ monocyte/macrophage possesses reduced antigen-presenting potential during HTNV infection (43).

Beyond the functional changes in specific monocyte subsets, a key question in hantavirus immunopathogenesis is why HTNV often causes lethal HFRS in humans but establishes a long-term asymptomatic carrier state in its natural rodent host, the striped field mouse. Ma et al. provided mechanistic evidence that different macrophage responses may contribute to distinct infection outcomes in humans and rodents. The key difference does not lie in viral replication itself, as both rodents and humans support HTNV replication, but rather in the differential capacity of host macrophages to regulate inflammation. In humans, HTNV-infected monocytes and macrophages exhibit persistent activation of a pro-inflammatory M1-like phenotype, driving a TNF-α centered cytokine storm that contributes to HFRS pathogenesis. In the natural rodent host, however, macrophages undergo only transient activation in the early stage of infection. This is rapidly suppressed by a negative feedback mechanism mediated by a rodent-specific lncRNA, which inhibits p65 phosphorylation and induces late-phase inactivation of M1 macrophages, thereby preventing significant tissue damage and maintaining an asymptomatic carrier state. Human macrophages lack this negative feedback mechanism and therefore remain persistently activated toward the M1 phenotype, which is a key factor underlying the cytokine storm in HFRS (10). Collectively, these findings highlight the complex roles of monocytes and macrophages in HFRS pathogenesis and offer a new perspective for understanding species-specific pathogenicity.

## Dendritic cells drive both protective antiviral immunity and immunopathological damage in HTNV infection

DCs are professional antigen-presenting cells and are broadly classified into two major lineages: plasmacytoid DCs (pDCs) and classical DCs (cDCs). pDCs originate from lymphoid precursors and often exhibit an intrinsic resistance to the majority of viral pathogens (44, 45). In contrast, cDCs are mainly myeloid-derived antigen-presenting cells that initiate adaptive immune responses. It is well established that DCs act as “fire accelerants” in HTNV pathogenesis (11).

HTNV infection profoundly reprograms DCs, driving them toward a state of strong maturation and activation (12). After recognizing viral components through PRRs such as RIG-I, monocyte-derived DCs upregulate MHC class I and class II molecules together with co-stimulatory CD80 and CD86, thereby enhancing their ability to present endogenous viral antigens to CD8^+^ T cells and exogenous viral antigens to CD4^+^ T cells via the MHC class I and class II pathways, respectively (12, 46). In addition to these conventional pathways, these matured DCs also enhance cross-presentation, a process by which exogenous antigens are loaded onto MHC class I molecules for presentation to CD8^+^ T cells, thereby expanding the range of antigens that can trigger a CD8^+^ T cell response (46, 47). At the same time, upregulation of the chemokine receptor CCR7 allows these mature DCs to migrate from peripheral tissues to draining lymph nodes (11), where they initiate robust adaptive immune responses. Moreover, these DCs markedly upregulate the inhibitory ligands PD-L1 and PD-L2 (48). Normally, PD-L1 upregulation would suppress T cell activation by binding to PD-1 on T cells. However, studies have shown that this inhibitory effect is blocked, as CD80 on the DC surface can bind to PD-L1 on the same DC, effectively occupying PD-L1 and preventing it from engaging with PD-1 on T cells. As a result, the brake on T cell activation is largely relieved (49). Consistent with these observations, levels of the soluble co-stimulatory molecule sCD86 and the soluble inhibitory molecules sPD-L1 and sPD-L2 are all elevated in the sera of patients with HFRS (48), yet T cell activation remains robust (14, 50).

Beyond these direct effects on DC maturation and T cell activation, the functional properties of DCs also contribute to bystander activation of immune cells. HTNV-infected inflammatory DCs, which highly express CD14, can promote rapid activation of bystander CD8^+^ T cells in a T cell receptor (TCR)-independent manner (48, 51). These bystander-activated T cells show innate-like cytolytic activity and secrete pro-inflammatory cytokines such as interferon-γ (IFN-γ), which may contribute to tissue injury (52, 53). In addition, hantavirus-induced bystander activation also affects NK cells (54). These bystander-activated immune cells in turn activate other uninfected DCs, leading to enhanced cross-presentation of viral antigens and the generation of virus-specific, TCR-dependent adaptive immune responses (54, 55).

Notably, HTNV-infected DCs may also act as “Trojan horse” carriers. Lung-resident cDCs, whose dendritic projections extend into the airway lumen through epithelial tight junctions, are among the first cells to encounter inhaled HTNV and become infected. A proposed model suggests that HTNV may hijack​ these infected DCs to transport replicating virus to endothelial cell monolayers, thereby delivering the infection to its principal target cells. However, it remains unclear how HTNV travels from the lungs to the endothelial barrier *in vivo*, and this Trojan horse mechanism remains a plausible hypothesis rather than a fully proven process (11). In summary, DCs in HTNV infection produce two functionally divergent immune outcomes. On the one hand, HTNV-induced mature DCs promote virus-specific immunity through enhanced antigen presentation and costimulation, along with relief of PD-L1-mediated T cell suppression. On the other hand, HTNV-infected DCs drive bystander immune amplification and may serve as “Trojan horses” for viral dissemination. The balance between these processes determines whether the host clears the virus or develops immunopathology.

## Dynamic regulation of NK cell subsets during HTNV-induced HFRS

NK cells play a complex role in HTNV infection, characterized by heterogeneity in subset distribution, receptor expression, and effector functions. Regarding the total abundance of NK cells, the literature presents conflicting findings. For instance, some studies have reported a significant increase in peripheral blood NK cell counts throughout the course of HFRS, with a pronounced peak during the oliguric phase, suggesting a robust mobilization of innate immunity (17). In contrast, other studies observed no significant change in the overall proportion of NK cells (14). These discrepancies may reflect differences in analytical approaches, patient populations, or disease severity.

At the subset level, Li et al. found that the number of CD56^dim^CD16^+^ NK cells, traditionally considered the main cytotoxic subset, did not show statistically significant fluctuations (17). However, our previous study revealed that the frequency of this subset during the febrile phase was correlated with disease severity, pointing to its potential utility as an early biomarker for risk stratification (14). In contrast to the relative stability of this cytotoxic subset, the predominantly immunoregulatory CD56^bright^CD16^−/+^ and CD56^−^CD16^+^ NK cell subsets showed a marked increase, particularly during the oliguric phase (17). Furthermore, another study observed that CD56^+^CD16^-^ NK cells began to expand as early as the acute phase of infection and remained high through the recovery phase (16), implying their involvement in immune regulation and tissue repair throughout the disease course.

The effector functions of NK cells are finely regulated by the balance of activating and inhibitory receptors on their surface. In patients with HTNV-induced HFRS, studies have reported a decrease in the expression of the inhibitory receptor NKG2A, alongside an increase in the activating receptor NKG2D (17). This shift in receptor expression may help relieve inhibitory signaling and promote antiviral immune responses. Beyond these changes, a more comprehensive analysis has revealed another notable feature of NK cell receptor dynamics. Several activating receptors, including NKp30, NKp44, and NKp46, as well as inhibitory receptors such as KIR2DL2/3, and the dual-function receptor KIR2DL4, remained persistently overexpressed beyond the acute phase (16). This sustained immunoregulatory mechanism enables NK cells to maintain their antiviral capacity over an extended period. Interestingly, despite the overall downregulation of the inhibitory receptor NKG2A, Su et al. found that the proportion of the CD56^dim^CD16^+^NKG2A^+^ NK cell subset did not change significantly during HTNV infection (14). Single-cell transcriptomic studies by Xue et al. further revealed that CD56^dim^CD16^+^NKG2A^+^ NK exhibited an activated and proliferative state during the acute phase and were capable of secreting effector cytokines such as TNF-α and IFN-γ (28).

The complex receptor landscape described above is actively shaped by HTNV through at least two different mechanisms. First, to relieve inhibition, HTNV-derived peptides, such as NP5 and GP7, can stably bind to the HLA-E*0103 molecule, yet the resulting complex is not effectively recognized by the CD94/NKG2A receptor, thereby relieving the inhibitory signal and enhancing NK cell antiviral activity (28). Second, to enhance activation, HTNV infection upregulates the expression of multiple ligands on endothelial cells, including ICAM-1, VCAM-1, classical HLA class I molecules, and HLA-G. The interaction of these ligands with their corresponding receptors on NK cells enhances NK cell-mediated secretion of cytokines and chemokines (16). For example, HLA-G can interact with KIR2DL4 on NK cells, further stimulating NK cell proliferation and cytokine secretion (16). IFN-γ secreted by NK cells can activate macrophages, and the IL-12 produced by activated macrophages in turn promotes NK cell activation, forming a highly efficient positive feedback loop for antiviral immunity (56). However, such potent immune activation must be tightly controlled. Studies have shown that decreased plasma levels of soluble HLA-G (sHLA-G) are associated with overactivation of T cells and NK cells, which may exacerbate vascular permeability and thereby contribute to or aggravate the typical pathological features of HFRS (16). Taken together, the functional remodeling and dysregulation of NK cells, along with their regulatory effects on other immune cells, collectively shape the immunopathological process of HTNV-induced HFRS.

## Neutrophils as a double-edged sword between antiviral defense and immunopathology in HTNV infection

Neutrophils are the most abundant innate immune cells and can mediate pathogen defense by multiple processes (57, 58). Upon reaching the infection site, neutrophils eliminate invading microbes by producing reactive oxygen species (ROS) and releasing antimicrobial proteins, such as MPO and neutrophil elastase (NE) in a process of degranulation (59, 60). In addition, neutrophils also release NETs, a fibrous network composed of a double-stranded DNA (dsDNA) backbone and coated with histones and granule molecules like MPO and NE, with the functions of entrapping and killing pathogens (61, 62).

However, uncontrolled neutrophil activation may promote immunopathology in HFRS. In patients with HTNV-induced HFRS, research findings show that plasma MPO levels are significantly elevated during the acute phase compared to healthy controls, and that MPO levels in the critical or severe group are significantly higher than those in the moderate/mild group (18). Plasma MPO levels positively correlate with WBC count, neutrophil count, BUN, uric acid (UA), and Cr, but negatively correlate with PLT count (18). Similarly, Raftery et al. detected NETs in kidney biopsies from hantavirus-infected patients, providing direct histological evidence of NET deposition in renal tissue. Furthermore, based on serological findings, specifically elevated circulating NET components and intact DNase I activity, they proposed that excessive NET formation may overwhelm normal NET clearance, leading to systemic NET overflow (29). This systemic NET release exposes self-DNA to immune cells in lymph nodes, activating autoreactive memory B cells to produce autoantibodies that form immune complexes with NETs. These immune complexes subsequently deposit in the kidney, activate complement, and contribute to renal damage (29). Beyond excessive degranulation and NET formation, activated neutrophils that have adhered to and transmigrated across the endothelium can release TNF-α (63), which directly increases vascular permeability by inducing the internalization and degradation of vascular endothelial (VE)-cadherin (64, 65). Also, activated neutrophils can secrete VEGF, a key mediator of VE-cadherin degradation, thereby further compromising the endothelial barrier (58). Additionally, neutrophil-platelet interactions may also contribute to HFRS pathology. Li et al. showed that neutrophil-platelet aggregates (NPAs) are significantly increased in HTNV-HFRS patients during the acute phases compared with healthy controls (18). This elevated NPA formation likely reflects abnormal platelet activation and consumption, leading to a reduction in circulating free platelets, which is a key factor underlying the hemorrhagic manifestations of HFRS. Also, these NPAs may actively participate in vascular inflammation and tissue injury through platelet-neutrophil crosstalk (18).

Animal studies further support the pathogenic potential of dysregulated neutrophil responses. In HTNV-infected SCID mice, the onset of pulmonary edema is accompanied by increased neutrophils in both peripheral blood and lung tissue. Neutrophil depletion mediated by anti-Gr-1 monoclonal antibody significantly suppresses HTNV-induced pulmonary vascular hyperpermeability and effectively reduces the severity of pulmonary edema (19). Similarly, infection with the highly pathogenic KHF5 strain of HTNV induces weight loss, renal hemorrhage, and a marked increase in neutrophils, accompanied by enhanced transcriptional signatures of neutrophil activation (66). Collectively, these findings indicate that neutrophils may serve as double-edged innate effectors during HTNV infection: appropriate activation supports early antiviral defense, whereas excessive or sustained activation may aggravate vascular leakage, platelet abnormalities, and organ injury.

## Protective and pathogenic CD8^+^ T cell responses in HTNV infection

CD8^+^ T cells are central effectors of the adaptive immune response following HTNV infection. Liu et al. reported that during the febrile phase, the proportions of CD3^+^ total T cells and CD3^+^CD8^+^ T cells were elevated, whereas the proportion of CD3^+^CD4^+^ T cells and the CD4^+^/CD8^+^ ratio were significantly decreased (67). Our earlier study also confirmed that the proportion of CD8^+^ T cells in the peripheral blood of patients with HFRS peaked during the febrile and oliguric phases, followed by a gradual recovery in the convalescent phase (14). These findings indicate that HTNV infection induces a cytotoxic T cell-dominated antiviral immune response.

At the subset level, HTNV infection drives T cell differentiation toward an effector phenotype. During the acute phase, CD8^+^ Tem cells were significantly expanded, whereas the proportion of CD8^+^ Tnaïve cells decreased (14). Notably, the proportion of CD8^+^ Tem cells in severe patients during the febrile phase was significantly higher than that in mild patients, suggesting that this parameter could serve as an early risk stratification marker (14). In addition, Zhang et al. reported an increase in CD4^+^CD8^+^ double-positive T cells in the peripheral blood of HFRS patients. They further demonstrated that these cells belonged to the CD8^+^ T cell lineage, exhibited high expression of granzyme B and CD107a, and possessed a more diverse T cell receptor repertoire, reflecting a clear CD8 lineage signature with antiviral function (30).

Functionally, acute phase CD8^+^ T cells displayed a markedly activated state. Our earlier study revealed that CD38^+^CD8^+^ T cells were significantly increased during the febrile and oliguric phases and gradually decreased during the convalescent phase (14). On the protective side, Tang and colleagues found that HLA-E-restricted HTNV-specific CD8^+^ T cells were more abundant in patients with mild to moderate disease than in those with severe disease. These cells produced stronger IFN-γ and granzyme B responses, and their frequency was negatively correlated with plasma viral load (20). These findings underscore that high-quality, virus-specific CD8^+^ T cell responses effectively limit viral replication and are associated with better clinical outcomes. Similarly, IFN-γ-producing T cell responses to HTNV nucleocapsid protein were induced during acute infection, but were markedly lower in patients with severe/critical HFRS compared with mild/moderate cases, suggesting that deficient early virus-specific cellular immunity may contribute to severe disease (68). Consistently, the level of HTNV-specific CD8^+^ T cells has been reported to be closely associated with disease progression. It increases significantly during the febrile phase and drops markedly during the hypotensive phase (69). Moreover, this cell level is negatively correlated with BUN and Cr levels and is inversely associated with proteinuria severity, further supporting its role in viral control. On the other hand, activated CD8^+^ T cells also contribute to immunopathological damage. A large number of activated CD8^+^ T cells release IFN-γ, TNF-α, and cytotoxic granules, attacking vascular endothelial cells expressing viral antigens, thereby promoting inflammation amplification, increased vascular permeability, and renal injury (8, 70).

Beyond their functional activation, CD8^+^ T cells also undergo exhaustion and apoptosis during HTNV infection. Our study found increased PD-1 expression on CD8^+^ T cells during the acute phase, suggesting that some CD8^+^ T cells may gradually enter an exhausted state (14). Consistent with this, serum levels of soluble TIM-3, LAG-3, and PD-1 were all significantly elevated in the early stage of HFRS. Among these molecules, soluble TIM-3 positively correlated with blood urea levels, soluble LAG-3 negatively correlated with PLT counts, and soluble PD-1 positively correlated with Cr and lactate dehydrogenase (LDH) levels (14, 50). In addition, HTNV infection can induce T cell apoptosis through activation induced cell death (AICD), as evidenced by increased caspase-3 activity, suggesting that AICD may also contribute to the pathogenesis of HFRS (24).

In addition to these regulatory pathways, HTNV can directly modulate CD8^+^ T cell function through distinct mechanisms. For instance, HTNV can directly infect CD8^+^ T cells and complete a full replication cycle, with progeny virus released via microvesicle transport. More importantly, the number of infected CD8^+^ T cells positively correlated with disease severity (22). Moreover, IL-15 secreted by endothelial cells can induce bystander activation of Epstein-Barr virus or cytomegalovirus specific CD8^+^ T cells, which then exert cytotoxic effects on HTNV-infected endothelial cells via the NKG2D receptor, revealing a critical role of the IL-15/NKG2D axis in the immunopathology of HFRS (71).

These insights into CD8^+^ T cell responses have important implications for vaccine development. Ma et al. found that the HLA-A02 restricted CTL epitope FA9 derived from the HTNV nucleoprotein exhibited multifunctional activity and induced a potent CD8^+^ T cell immune response, thereby mediating effective protective responses (31). Tang et al. identified seven HLA-A0201-restricted CTL epitopes from HTNV glycoproteins, among which the immunodominant epitope LL9 effectively induced protective CTL responses in HLA-A2.1/Kb transgenic mice and inhibited viral replication in target organs (72). Another study designed an HLA-A02-restricted HTNV multi-antigen peptide, which significantly stimulated CD8^+^ T cell proliferation and IFN-γ secretion. This peptide demonstrated superior efficacy compared with single CTL epitopes and represents an important direction for future vaccine development (73).

Collectively, CD8^+^ T cells exert a dual and critical role during HTNV infection. On the one hand, virus specific CD8^+^ T cells and their subsets exert antiviral protective effects through cytokine production and cytotoxic activity. On the other hand, hyperactivated T cells can drive immunopathological tissue damage. Additional mechanisms, such as direct viral infection of T cells, AICD, and T cell exhaustion, further shape the balance of the immune response (**Table 1**). These mechanistic insights have, in turn, guided the development of multi-epitope vaccines aimed at that eliciting potent protective CTL responses.

**Table 1:** Protective and pathogenic roles of CD8^+^ T cells in HTNV-induced HFRS.

Protective role	Immunopathological role
CD3^+^CD8^+^ T cells: peaked during febrile/oliguric phases and gradually recovered in convalescence, reflecting a cytotoxic T cell-driven antiviral response (14, 67).	T-cell hyperactivation attacks endothelium: Large numbers of activated CD8^+^ T cells release IFN-γ, TNF-α, and cytotoxic granules, causing vascular leakage and renal injury (8, 70).
CD4^+^CD8^+^ double positive T cells: Belong to CD8 lineage, exhibit high levels of granzyme B and CD107a, possess a more diverse TCR repertoire, reflecting potent antiviral functions (30).	T-cell exhaustion: PD-1 upregulation in acute phase; soluble TIM-3 positive correlation with blood urea, LAG-3 negative correlation with platelet count, PD-1 positive correlation with Cr and LDH (14, 50).
HLA E–restricted CD8^+^ T cells: More abundant in mild to moderate patients, produce strong IFN-γ and granzyme B; frequency negatively correlated with viral load (20).	T-cell apoptosis: HTNV infection induce T cell apoptosis through activation induced cell death (AICD) (24).
HTNV nucleocapsid-specific IFN-γ^+^ T cells: induced during acute infection; significantly lower in severe/critical patients, suggesting early deficiency may promote severe disease (68).	HTNV-infected CD8^+^ T cells: support full virus replication; progeny virions released via microvesicles; number of infected cells positively correlates with disease severity (22).
HTNV-specific CD8^+^ T cells: HTNV-specific CD8^+^ T cells negatively correlate with BUN, Cr, and proteinuria, exerting viral control (69).	Bystander activation: Endothelial IL-15 induces EBV/CMV-specific CD8^+^ T cells to kill HTNV-infected endothelial cells (71).

## CD4^+^ T cell dynamics and functional implications in the context of HTNV infection

During HTNV infection, CD4^+^ T cells undergo significant dynamic changes and have multiple roles in the development of HTNV-induced HFRS. In the febrile and oliguric phases, the proportion of CD3^+^CD4^+^ T cells in peripheral blood is significantly lower than that in healthy controls and gradually recovers during the polyuric and convalescent phases (14, 67). However, absolute CD3^+^CD4^+^ T cell counts do not differ significantly between patients at any disease stage and healthy controls, nor do they vary markedly between mild and moderate cases (74). This indicates that the early inversion of the CD4^+^/CD8^+^ ratio is not driven by a reduction in CD4^+^ T cell numbers. Nonetheless, severe patients show a significantly lower frequency of CD4^+^ Tcm during the oliguric phase, suggesting that memory T cell homeostasis is perturbed in severe disease (14).

Functionally, CD4^+^ T cells are involved in antiviral immunity primarily through cytokine secretion. HTNV glycoprotein-specific CD4^+^ T cells are capable of mounting a potent Th1 response characterized by multifunctional cytokine production. The magnitude of this response is inversely correlated with plasma viral RNA load, underscoring its importance in viral control (75). The differentiation status of these cells also differs according to disease severity. In patients with mild to moderate disease, HTNV glycoprotein-specific CD4^+^ T cells predominantly display an effector memory (CCR7^−^CD45RA^−^) and highly differentiated (CD27^−^CD28^−^) phenotype. In contrast, those with severe or critical illness are marked by a central memory phenotype (CCR7^+^CD45RA^−^) and an early differentiated state (CD27^+^CD28^+^) (75). At the systemic level, both Th1 and Th2-type cytokines were markedly elevated during the first week of illness compared with controls. IL-2 peaked at four weeks and remained elevated at twelve weeks, while IL-10 was persistently high throughout the disease course. Notably, IL-10 levels during the convalescent phase were significantly higher in moderate patients than those in mild patients (74). Together, these results indicate that the cytokine secretion profile related to CD4^+^ T cell responses is strongly associated with disease severity, underscoring its utility as a key immunological parameter for assessing disease progression and prognosis.

In addition to their regulatory functions, CD4^+^ T cells are also directly targeted by HTNV. The virus can enter human CD4^+^ T cells via T cell immunoglobulin and mucin domain -containing protein 1 (TIM-1) (23). Once infected, these cells show impaired function, including reduced secretion of Th1 and Th2-type cytokines (75). Since these cytokines are critical for antiviral effector function, such dysfunction may lead to insufficient viral clearance. Moreover, as CD4^+^ T cells are indispensable for CD8^+^ T cell cytotoxicity, B cell activation, and plasma cell differentiation, their functional impairment can substantially compromise the quality of both cellular and humoral immune responses.

From a vaccine perspective, MHC class II restricted epitopes derived from the HTNV nucleoprotein have shown broad immunoreactivity. They can effectively induce CD4^+^ T cell responses, thus providing a theoretical basis for epitope based vaccine design (76). Taken together, these findings elucidate the role of CD4^+^ T cells in the immunopathogenesis of HFRS, highlighting their dual function as essential immune regulators and direct targets of the virus.

## Dynamic changes and functional heterogeneity of nonclassical T cells in HTNV-induced HFRS

Nonclassical T cells, primarily including mucosal-associated invariant T (MAIT) cells, natural killer T (NKT) cells, and γδ T cells, serve as crucial bridges linking innate and adaptive immunity (77). Their roles in HTNV infection are gradually being elucidated.

MAIT cells are characterized by high surface expression of the C-type lectin CD161 and constitute approximately 10% of circulating T cells in humans (78). Functionally, they play a protective role in HTNV infection. Zhang et al. found that patient-derived CD8^+^ MAIT cells can produce IFN-γ and granzyme B and inhibit HTNV replication in vascular endothelial cells *in vitro* (21). Further investigation confirmed that IL-18 signaling is indispensable for this activation, as blockade of IL-18 significantly attenuates MAIT cell effector function (21). Consistently, in a MAIT cell-deficient mouse model, HTNV infection resulted in increased viral load and aggravated pathological damage, directly demonstrating the protective role of MAIT cells in antiviral defense (79). However, despite their protective function, the number of MAIT cells declines during the febrile and oliguric phases (14). Further mechanistic investigations have revealed a core mechanism underlying MAIT cell loss, in which viral infection induces endoplasmic reticulum calcium overload, subsequently activating the IRE1α-mediated endoplasmic reticulum stress pathway and ultimately triggering MAIT cell pyroptosis (79). Administration of an IRE1α inhibitor significantly reduced the level of MAIT cell pyroptosis, supporting the central role of this pathway (79). Despite their overall reduction, the remaining MAIT cells exhibit persistent activation evidenced by upregulated CD38 (14). Therefore, appropriately regulated MAIT cell activation contributes to viral clearance, whereas persistent or excessive stimulation may promote MAIT cell pyroptosis, thereby compromising host defense.

NKT cells are innate-like T lymphocytes that co-express CD3 and CD56 (80). Upon stimulation, these cells secrete IFN-γ and IL-4, contributing to antiviral immune responses (80). Our longitudinal analysis of HTNV-infected patients revealed that although the proportion of NKT cells remained stable throughout the course of HFRS, they were significantly activated during the febrile and oliguric phases, as evidenced by increased CD38 expression (14). γδ T cells constitute a distinct lymphocyte subset that expresses a γδ T cell receptor (TCR) rather than the conventional αβ TCR and plays important roles in antiviral defense (81, 82). We further observed that while the proportions of Vδ1 and Vδ2 subsets of γδ T cells did not change significantly, the proportion of γδ^+^ effector memory T cells (Tem γδT) decreased, whereas that of γδ^+^ naïve T cells (Tnaïve γδT) increased, suggesting that HTNV infection can modulate the differentiation status of γδ T cells (14). Currently, studies on NKT and γδ T cells are largely limited to alterations in their proportions and phenotypic profiles, and direct evidence regarding their cytokine secretion, cytotoxic activity, and relationship with disease severity remains lacking.

## B cell dynamics and humoral immunity during HTNV infection

B cell mediated humoral immunity is a core component of the adaptive immune response following HTNV infection. Regarding overall cell counts and subpopulations, longitudinal studies revealed that the total B cell proportion did not change significantly (14). Of note, in the febrile phase, plasmablasts (PBs) exhibited a marked expansion of approximately 200-fold (14). Meanwhile, antibody secreting memory B cells (ASMs) became significantly enriched, while naïve and memory B cell subsets decreased (26). These findings suggest that B cells undergo polyclonal differentiation from a resting state toward an antibody-secreting phenotype. After the serum antibody titer increased fourfold, the key transcription factors driving B cell differentiation into antibody-secreting cells, including PRDM1, IRF4, and XBP1, showed a significant decline in transcript levels (25). This decline occurred in parallel with the rapid reduction of PB numbers following their early peak (14, 26). This pattern indicates that in an effective humoral immune response, the expression of these transcription factors is tightly controlled to ensure timely contraction of plasma cell responses after antibody secretion. In contrast, transcription factors that help maintain normal B cell identity and function, such as Pax5, IRF8, and Bcl6, are upregulated during the convalescent phase (25). This upregulation is particularly pronounced in patients with moderate HFRS, suggesting that B cells can more effectively return to a resting state after the immune response.

In terms of antibody effector function, neutralizing antibodies are key protective molecules produced by B cells. For example, a monoclonal antibody named HTN-Gn1, obtained from animals immunized with recombinant HTNV Gn protein, specifically recognizes the surface-exposed Gn epitope, confirming that Gn is a key protective antigen capable of inducing neutralizing antibodies (27). Beyond neutralization, antibody function is also significantly modulated by glycosylation of the Fc region. Quan et al. conducted a glycomic analysis of the IgG Fc region in HFRS patients and found that core fucosylation increased during the convalescent phase, whereas the level of bisecting N-acetylglucosamine decreased (26). Importantly, these changes are closely linked to the differentiation state of B cells. The proportion of the ASM subset showed a positive correlation with the levels of galactosylation and sialylation in the IgG Fc region, while the proportion of the PB subset correlated positively with sialylation levels (26). Single-cell transcriptomic data further demonstrated that multiple glycosylation-related genes are enriched in the ASM and PB subsets (26). These findings indicate that the effector functions of antibodies are influenced by Fc glycosylation, which is strongly associated with the differentiation state of B cells.

Beyond the functional antibody features, the underlying B cell receptor (BCR) repertoire itself undergoes profound remodeling. High throughput sequencing showed that the BCR clonal expansion was more enhanced in HFRS patients than in healthy controls. Also, shifted in the usage frequencies of certain IGHV and IGHJ genes indicated extensive BCR repertoire remodeling (83). Notably, the diversity of immunoglobulin genes increased significantly during the convalescent phase, and this increase was more pronounced in patients with moderate HFRS than in those with severe HFRS (25). This further supports the critical role of a prompt and efficient humoral immune response in disease recovery.

Notably, patients with varying disease severity exhibited distinct patterns of B cell response. In moderate cases, B cell activation was rapid and robust during the first two weeks, with plasmablasts peaking in the first week and declining thereafter, while activated and memory B cells expanded markedly. In contrast, severe patients exhibited delayed and diminished B cell responses with restricted BCR repertoire diversification, and B cell associated transcription modules were negatively enriched. Additionally, severe patients displayed marked upregulation of pro-inflammatory gene sets and cytokines, alongside elevated anti-inflammatory cytokines, suggesting a hyperinflammatory state with compensatory anti-inflammatory responses (25). These findings suggest that a breakdown of the balance between B cell activation and inflammatory control contributes to the delayed viral clearance and poor outcome in severe HFRS (20).

## Conclusion and discussion

**Figure 1:**
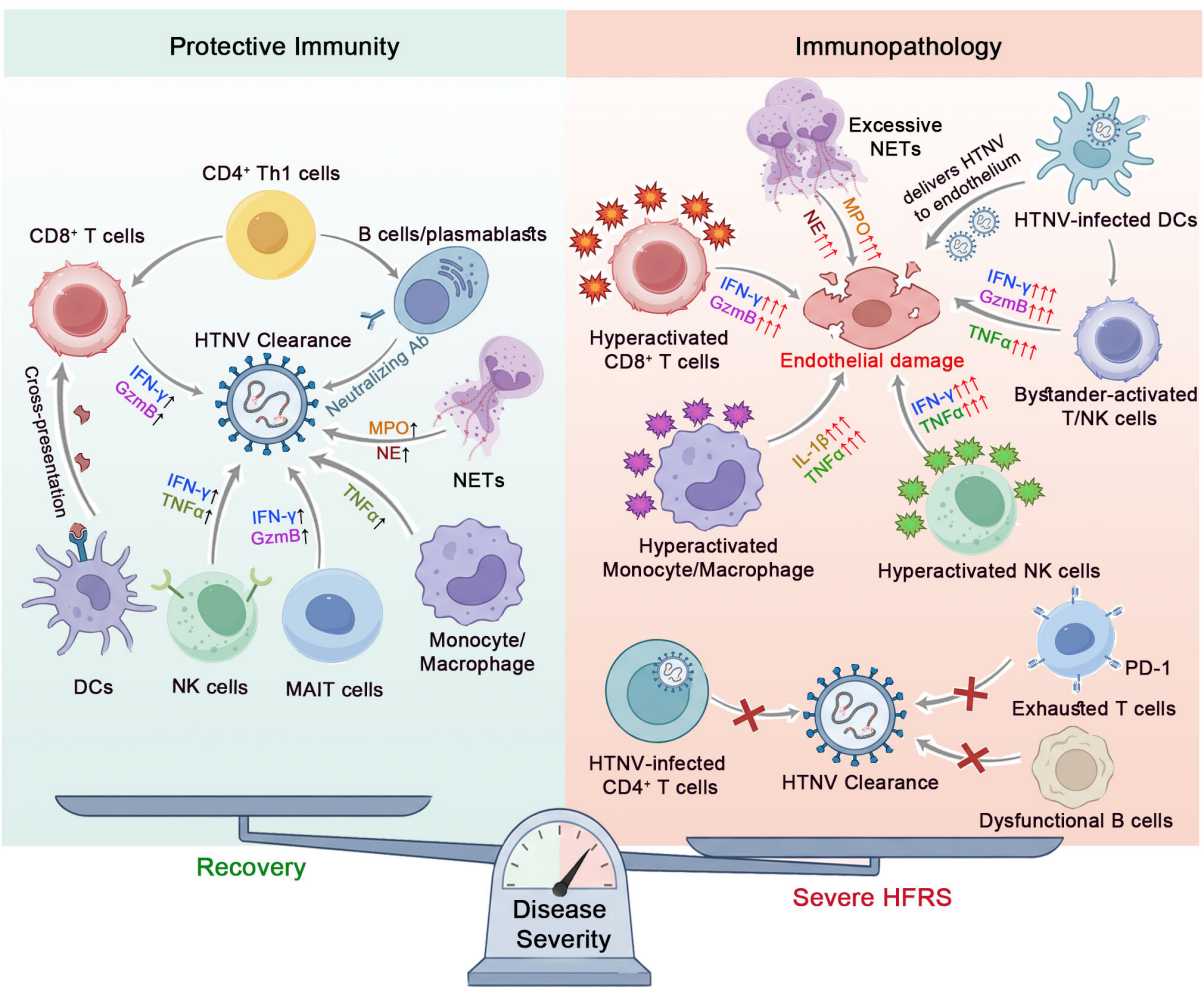
Schematic illustration of the dual-edged sword effect of the immune cells following HTNV infection. This diagram depicts the dynamic balance between protective immunity and immunopathological damage, and this equilibrium ultimately determines disease severity. The left panel highlights the protective antiviral mechanisms mediated by CD8^+^ T cells, CD4^+^ Th1 cells, B cells/plasmablasts, MAIT cells, monocytes/macrophages, neutrophils, DCs and NK cells, which collaborate to clear the virus and promote host recovery. The right panel illustrates the immunopathological mechanisms. On the one hand, overactivated CD8^+^ T cells and NK cells, hyperactive monocytes/macrophages, excessive NET formation and HTNV-infected DCs elicit endothelial injury, resulting in vascular leakage and tissue damage. On the other hand, HTNV-infected CD4^+^ T cells, exhausted CD8^+^ T cells, and dysfunctional B cells impair virus clearance. The balance between protective immunity and immunopathology dictates disease outcome: protective immunity leads to recovery and viral elimination, while immunopathology results in severe HFRS.

This review systematically describes the dynamic changes and functional remodeling of major immune cells during HTNV infection, including monocytes and macrophages, DCs, NK cells, neutrophils, T cells, and B cells. It highlights the dual role of immune cells in HFRS. The immune cells are both the main factors in controlling viral replication and one of the major contributors to pathological damage through their dysregulation (**Figure 1****).**

Monocytes and macrophages are essential for initiating antiviral responses (8, 38). However, HTNV infection drives human macrophages toward a persistent pro-inflammatory M1-like phenotype, promoting a TNF-α-centered cytokine storm contributing to HFRS pathogenesis (10). DCs are critical for initiating adaptive immunity as they present viral antigens to activate T cells (12, 46). However, HTNV-infected DCs can also serve as “Trojan horses,” directly transferring the virus to endothelial cells (11). Also, these infected DCs drive widespread bystander activation of both T cells and NK cells in a TCR-independent manner, an innate-like response that can exacerbate tissue damage (48, 51, 52, 54). NK cells show a similar functional division. While their activating receptors are upregulated to promote antiviral activity (16, 17, 28), persistent overexpression of these receptors can cause excessive activation, directly contributing to immunopathology (16, 28). Neutrophils can mediate early antiviral defense through degranulation and NET formation (59, 60), yet uncontrolled neutrophil activation can promote vascular leakage and renal injury through excessive NET formation and neutrophil-platelet aggregates (18, 29). CD8^+^ T cells also have a dual function. They exert potent antiviral effects through IFN-γ and granzyme B (20). Nevertheless, their excessive activation and bystander cytotoxicity can trigger vascular leakage and renal injury (71). Similarly, although CD4^+^ Th1 responses contribute to viral control, direct infection by HTNV via the TIM-1 receptor impairs their helper functions, thereby diminishing the overall coordination of the adaptive immune response (23, 75). B cell mediated humoral immunity also plays a dual role during HTNV infection (14, 25, 26). In mild to moderate HFRS patients, PBs expand and contract rapidly, reflecting efficient protective function (25, 26). In severe patients, B cells fail to fulfill their protective functions due to functional impairment. Overall, these results highlight an immunological duality where the immune processes necessary for eliminating viruses can, if not properly regulated, lead to the severe symptoms seen in HFRS.

Despite these advances, several critical questions remain unanswered. First, most existing studies rely on peripheral blood samples, changes in the number and ratio of immune cells in peripheral blood might not accurately reflect immune status in the kidney, the primary target organ (84, 85). Currently, the limited but valuable studies using renal biopsies and animal models have begun to delineate the intrarenal immune landscape of HTNV-induced injury (86). Immunohistochemical analyses of renal biopsies from HFRS patients revealed dense interstitial infiltration of lymphocytes and monocytes/macrophages, with a predominant CD8^+^ T cell presence particularly in the renal medulla (87). In HTNV-infected mouse models, CD8^+^ T cell depletion completely abrogated renal hemorrhagic lesions despite unchanged viral loads, establishing CD8^+^ T cells as indispensable drivers of renal pathology, with virus-specific cytotoxic T lymphocytes enriched in renal tissue at far higher proportions than in the spleen. Furthermore, disease outcome may be determined not only by viral load but also by the balance between infected target cells and virus-specific CD8^+^ T cells (88). In addition to these T cell-mediated mechanisms, recent evidence highlights the potential contribution of NETs to renal immunopathology. Through immunofluorescence analysis, NETs were detected in kidney biopsies from hantavirus-infected patients, with three out of four biopsy samples from four different patients testing positive, while all healthy kidney tissue controls remained negative (29). These intrarenal observations, though derived from a small number of studies, provide compelling evidence of active, localized immune effector processes within the kidney. Since directly obtaining renal tissue from HFRS patients presents clinical challenges, alternative approaches are required to bridge this gap. One feasible approach is to investigate the relationship between organ-specific injury markers and blood immune cell subsets, which may indirectly reflect changes in the tissue microenvironment. The use of animal models, particularly humanized mice or nonhuman primates, is an important complement. These models allow for the direct analysis of immune infiltration and cellular interactions within target organs under controlled conditions (89–92). Although such models cannot fully recapitulate the complexity of human HFRS, they provide an indispensable platform for validating the tissue relevance of findings derived from peripheral blood.

Second, current studies often describe the immune response as a linear and unidirectional causal chain. In this model, the virus activates immune cells, these cells exert effector functions, and excessive effects then lead to pathology. However, the true picture of HTNV infection is more complicated. There may exist a safety threshold for immune activation (93). Below this threshold, the immune response operates within a coordinated and self-limiting linear amplification zone, effectively clearing the virus. Once this threshold is exceeded, however, a cascade of positive feedback loops mediated by chemokines and cytokines may be triggered. This type of positive feedback is a hallmark of nonlinear, self-sustaining oscillatory dynamics, eventually leading to an uncontrolled immune storm. Theoretical models indicate that this nonlinear behavior arises from the dynamic interaction between immune cells and cytokines. When key parameters, such as the rate of cytokine production, are modified, the system may undergo a Hopf bifurcation, resulting in a shift from a steady state to periodic oscillations (93). Within this framework, NK cells, for example, are no longer simply protectors or destroyers, but are seen as key nodes in the network. Their condition and output are dependent on how strongly they interact with other nodes in the network, like DCs and macrophages, as well as on whether the system has crossed a critical threshold.

Third, it remains unclear whether the overactivation or sustained polarization of immune cells in severe HFRS may retain a pro-inflammatory memory beyond the convalescent phase, thereby increasing the risk of developing chronic inflammatory or autoimmune diseases in the future (94–96). Also, although CD8^+^ T cell responses directed against specific HLA-A*0201-restricted epitopes can effectively control the virus, the potential cross-reactivity between these epitopes and host tissues raises the concern that such responses may trigger autoimmune injury (97). These questions extend HFRS research from the acute phase to long-term prognosis and carry significant clinical implications.

Furthermore, most current studies focus on individual immune cell subsets. However, HTNV infection triggers a complex intercellular network response. The combined effects of synergy and antagonism across various cellular nodes ultimately shape the final output of the immune network. Importantly, HTNV infection is also accompanied by the emergence or reprogramming of several unconventional immune subsets. Examples include the emergence of CD4^+^CD8^+^ T cells (30), a notable increase in the CD56^−^CD16^+^ NK cells during the oliguric phase (17), and changes in the differentiation status of γδ T cells (14). These subsets may represent new network nodes created by the immune system as it tries to establish new homeostasis under extreme stimulation. Nevertheless, their functional significance and precise position within the immune cell network remain poorly understood. To thoroughly understand this complexity, future research should utilize high-resolution methods such as single cell RNA sequencing, single cell B cell receptor sequencing, and single cell T cell receptor sequencing (98–103). These strategies can systematically outline the composition, functional status, and clonal expansion patterns of immune cells at different stages of disease. These efforts will help the creation of a detailed map of dynamic immune cell interactions, thereby identifying the key cellular interaction modules that drive disease progression and outcome.

A key clue to understanding HFRS pathogenesis lies in the species difference. HTNV causes severe disease in humans but only an asymptomatic persistent infection in its natural reservoir, the striped field mouse (Apodemus agrarius). Previous studies have shown that human macrophages remain in sustained M1 polarization due to a lack of negative feedback mechanisms (10). This finding is important. It raises the question of whether the immune systems of humans and rodents follow fundamentally distinct regulatory architectures (104–106). For example, the missing negative feedback pathway in the human innate immune system may represent an evolutionary trade-off. In this scenario, tolerance is sacrificed to improve resistance, leading to increased hypersensitivity in the human host (107–109). Future research should systematically compare humans and rodents in several areas. This involves innate immune sensing, inflammasome activation, cytokine networks, and immunoregulatory cells. These efforts will not only help to clarify the pathogenic mechanisms of HFRS but also give general insights into diseases related to human-specific immunopathology.

Lastly, it is important to note that immune responses vary significantly even among hantaviruses responsible for HFRS (14, 110). These differences are probably caused by the unique features of the viral genome and structural proteins specific to each subtype. For example, sequence variations in the Gn and Gc glycoproteins may affect the virus’s ability to bind to immune cell receptors, the activation intensity of pattern recognition pathways, and the modulation of cytokine expression (111–113). Moreover, the harm to organs caused by hantaviruses is affected not only by the presence of crucial host cell receptors in the target organs (114, 115), but also by the innate immune microenvironment within those organs. Although low levels of viral antigen have been detected in the liver and intestine, these organs rarely suffer clinically significant injury in hantavirus disease, possibly due to the predominance of tolerogenic myeloid populations (116). Therefore, future research should not only aim to clarify how different hantavirus subtypes result in varying immunomodulatory effects but also seek to elucidate how the organ microenvironment acts as either an amplifier or a brake in this process.

In summary, this review highlights the dual role of the host immune response in HTNV infection, in which immune cells both combat the virus and contribute to disease. The shift from protective to harmful immunity involves complex cellular interactions and feedback loops. Future research should use systems-level approaches, including single-cell technologies and organ-level studies, to understand the mechanisms that shift the balance from effective defense to harmful immunopathology. This will enhance our understanding of HFRS and offer insights into inflammatory diseases and the balance between resistance and tolerance.
